# Developing a Socioeconomic Status Index for Chronic Disease Prevention Research in Canada

**DOI:** 10.3390/ijerph19137800

**Published:** 2022-06-25

**Authors:** Elham Khodayari Moez, Katerina Maximova, Shannon Sim, Ambikaipakan Senthilselvan, Roman Pabayo

**Affiliations:** 1 School of Public Health, University of Alberta, 3-300 ECHA, 11405 87 Avenue, Edmonton, AB T6G 1C9, Canada; ekhodaya@ualberta.ca (E.K.M.); smsim@ualberta.ca (S.S.); sentil@ualberta.ca (A.S.); pabayo@ualberta.ca (R.P.); 2 MAP Centre for Urban Health Solutions, Li Ka Shing Knowledge Institute, St. Michael’s Hospital, 209 Victoria St, Toronto, ON M5B 1T8, Canada; 3 Dalla Lana School of Public Health, University of Toronto, 155 College St Room 500, Toronto, ON M5T 3M7, Canada

**Keywords:** chronic disease prevention, socioeconomic status, index, smoking, Alberta’s Tomorrow Project

## Abstract

Capturing socioeconomic inequalities in relation to chronic disease is challenging since socioeconomic status (SES) encompasses many aspects. We constructed a comprehensive individual-level SES index based on a broad set of social and demographic indicators (gender, education, income adequacy, occupational prestige, employment status) and examined its relationship with smoking, a leading chronic disease risk factor. Analyses were based on baseline data from 17,371 participants of Alberta’s Tomorrow Project (ATP), a prospective cohort of adults aged 35–69 years with no prior personal history of cancer. To construct the SES index, we used principal component analysis (PCA) and to illustrate its utility, we examined the association with smoking intensity and smoking history using multiple regression models, adjusted for age and gender. Two components were retained from PCA, which explained 61% of the variation. The SES index was best aligned with educational attainment and occupational prestige, and to a lesser extent, with income adequacy. In the multiple regression analysis, the SES index was negatively associated with smoking intensity (*p* < 0.001). Study findings highlight the potential of using individual-level SES indices constructed from a broad set of social and demographic indicators in epidemiological research.

## 1. Introduction

In epidemiologic research, socioeconomic inequalities in health are some of the strongest and most robust findings. A strong socioeconomic gradient exists in chronic disease incidence, prevalence, morbidity and mortality [1,2,3,4]. Defined as one’s material and social standing relative to others [5], socioeconomic status (SES) also demonstrates strong, inverse and graded associations with the prevalence of unhealthy lifestyle behaviours. Indeed, socioeconomically disadvantaged adults are more likely to smoke, consume alcohol heavily, be physically inactive, have excess weight, and have poor dietary habits, compared to higher SES adults [6,7,8,9,10,11]. Studying socioeconomic inequalities in relation to chronic disease risk factors or outcomes is challenging and requires measuring SES based on individual- and/or neighbourhood-level characteristics [12,13]. Chronic disease researchers also often seek to account for the confounding effect of SES when isolating the effect of unhealthy lifestyle behaviours on chronic disease risk.

SES is a complex and multidimensional concept that encompasses many aspects, such as educational attainment, income adequacy, occupational prestige, employment status, gender, family structure and region of residence. All of these aspects can be represented by individual variables that are typically modeled separately, with education or income being used most commonly [12,13]. However, measuring SES using a single indicator may not adequately reflect the individuals’ complex socioeconomic conditions or circumstances. A single index synthesizing different socioeconomic aspects can be more meaningful for capturing the SES gradient in chronic disease risk factors or outcomes. In addition, a single SES index has been shown to better control for confounding than modelling different aspects separately [12].

One often-overlooked socioeconomic factor related to SES and not included in SES indices is gender. Gender refers to a social construct regarding culture-bound conventions, roles and behaviours for, as well as relations between and among, women and men or boys and girls [14]. The relationship between gender and SES is intertwined [15]. For example, wage trajectories and labour force participation rates over the lifespan are dependent on gender [15], which may in turn considerably impact health and well-being. By incorporating gender into socioeconomic indices, chronic disease researchers may achieve a more inclusive global measure of SES. Socioeconomic indices that do not include gender may not comparably capture inequalities in working and living conditions [16].

Many area-level indices have been constructed using census data for specific geographic areas to differentiate socioeconomic deprivation across neighbourhoods or communities. Area-level indices combine a number of geographic characteristics (% without high-school diploma; average income; % employed, % living alone; % separated, divorced, widowed; % single-parent families), using sophisticated variable reduction analytic techniques [17,18]. While area-level indices consider the contextual effects of SES on chronic disease risk factors or outcomes and are especially useful when individual-level data are absent, individual-level SES is the most direct measure of socioeconomic deprivation. However, individual-level SES indices are scarce and primarily rely on traditional average score approaches using a limited set of indicators (mainly education and income) rather than newer variable-reduction techniques based on a broad set of SES aspects that include occupational prestige and employment status, and incorporate important social and demographic characteristics such as gender, marital status, family structure and region of residence [19]. In Canada, the existing individual-level SES indices are dated and not used widely [20,21]. There is a need for a comprehensive and more-encompassing individual-level SES index that can be used to improve our understanding of the SES gradient in chronic disease risk factors and outcomes. To address this need, we constructed an individual-level SES index that incorporates a broad set of socioeconomic and demographic indicators, including educational attainment, income adequacy, occupational prestige, employment status, gender and region of residence. In the present paper, we describe the process of deriving the index and present an application of the derived SES index.

## 2. Materials and Methods

Between 2000 and 2008, Alberta’s Tomorrow Project (ATP) recruited 31,072 adults through a two-stage probability sample of non-institutionalised individuals aged 35–69 years living in Alberta, Canada, with no prior personal history of cancer other than non-melanoma skin cancer. Detailed protocols describing all data collection procedures have been previously published [22,23]. Socioeconomic and demographic characteristics were reported at baseline in the Core Questionnaire, which is also used in other cohorts comprising the national infrastructure platform of the Canadian Partnership for Tomorrow Project (CPTP). The Health Research Ethics Board of Alberta (HREBA)—Cancer Committee approved the ATP study procedures (HREBA.CC-17-0461) and current analyses (HREBA.CC-18-0101).

### 2.1. Indicators Used to Construct the SES Index

Years of schooling: Participants reported the highest level of educational attainment according to eight categories: none, elementary school; high school; trade, technical or vocation school, apprenticeship training or technical CEGEP (Collège d’enseignement général et professionnel); diploma from a community college, pre-university CEGEP or non-university certificate; university certificate below bachelor’s level; bachelor’s degree; and graduate degree (MSc, MBA, MD, PhD, etc.). We used the highest level of education completed combined with the age when this level of education was completed to construct the years of schooling as follows: no schooling (zero); elementary (1–6 years); high school (7–12 years), depending on the age of completion; a college diploma (14 years); graduates from trade, technical or vocational school or holders of any other degree below bachelor’s (15 years); bachelor’s degree (16 years) and graduate degree (17–20 years) [24].

Income adequacy: Participants reported a range of total gross household income according to eight categories: <$10,000; $10,000–$24,999; $25,000–$49,999; $50,000–$74,999; $75,000–$99,999; $100,000–$149,999; $150,000–$199,999; ≥$200,000. Participants also reported their marital status (married/living with a partner; divorced; widowed; separated; single, never married) and family structure, including children, parents and other persons living in or outside of the participant’s home, which was used to construct household size to determine the number of people (adults and children) supported by the household income. Each participant’s postal code was used to determine their geographic region of residence (metro, moderate metro influence, moderate urban influence, outside Alberta, rural, rural centre area, rural remote, urban). Eight categories of income adequacy were estimated from the ratio of total gross household income to the Statistics Canada low-income cut-off (pre-tax post-transfer for the reference year) for the applicable household size and community size group (based on the geographic region of residence). The ratios were ranked, and 8 percentiles were constructed within each Census Metropolitan Area, Census Agglomeration, or rural and small town area (outside Census Metropolitan Areas or Census Agglomerations) to account for regional differences in housing costs [4].

Occupational prestige: Participants reported their current main job title. The open-ended responses were coded according to the Statistics Canada’s National Occupational Classification (NOC) 2001 groups [20]. Occupational prestige was based on a ranking of occupational classes developed for Canada using the Frank and Goyder scale, which assigns a prestige score to 26 major Statistics Canada’s National Occupational Classification (NOC) groups [20].

Employment status: Participants reported their current employment status according to eight categories: full-time employed/self-employed; part-time employed/self-employed; retired; looking after home and/or family; unable to work because of sickness or disability; unemployed; doing unpaid or voluntary work; student. Employment status was then categorised into full-time, part-time, and other.

Gender: ATP surveyed participant biological sex (females and males). However, gender, a socially constructed “femaleness” or “maleness” in a society is inseparably interconnected and reciprocally influences sex [25]. For this investigation, we interpreted and discussed findings by integrating gender (women vs. men).

Smoking intensity: To demonstrate the index’s utility, we examined the association of the derived SES index with smoking intensity among past/current smokers and smoking history (past/current vs. never) among all participants. Smoking is one of the leading and most well-established risk factors for chronic disease [26,27], and there is a strong and persistent SES gradient in smoking, whereby those from lower SES backgrounds are more likely to participate in this behaviour [28,29,30]. Smoking intensity among past and current smokers was based on a number of cigarettes smoked per day and expressed in pack-years, defined as the number of packs of cigarettes (1 pack = 20 cigarettes) usually smoked per day multiplied by the number of years of smoking.

### 2.2. Statistical Analysis

Analyses were based on cross-sectional data from 17,371 participants that completed the Core Questionnaire at baseline and that had no missing data on all socioeconomic and demographic characteristics of interest in this study (as described above). To construct the SES index based on an expanded set of indicators (i.e., years of schooling, income adequacy, occupational prestige, employment status, gender), we used principal component analysis (PCA) with a polychoric correlation structure and varimax rotation to achieve orthogonality. We did not include marital status, family structure or region of residence to avoid overadjustment as this information is incorporated in the income adequacy variable. Selection of components to be retained was based on two criteria: the Kaiser criterion (eigenvalues ≥ 1) and individual proportion of variances per component explaining ≥10 percent of the overall variability. The final SES index was created by averaging the principal component scores (a numerical representation of the linear relationship between variables and the components) for each individual, according to the components retained. The range and distribution of individual inter-item correlations were examined to assess unidimensionality or the degree to which scale items assessed a single underlying factor or construct. We used Bartlett’s test of sphericity to detect any redundancy between variables that can be summarised with a fewer number of factors and the Kaiser–Meyer–Olkin (KMO) factor adequacy test to measure the common variation among the variables and evaluate whether the data were adequate for PCA. Only models with total explained variation greater than 0.6 and where all variables had KMO > 0.5 were considered. The SES index was then categorised into quintiles, where quintile 1 indicated lower SES and quintile 5 indicated higher SES. First, to assess how well the derived SES index aligned with its main components, we examined the distributions of three socioeconomic indicators (educational attainment, income adequacy percentiles, occupational prestige) for each SES quintile. Then, to demonstrate the utility of the SES index, we examined the associations of the SES index with smoking, an external factor, using multiple regression models, adjusted for age and gender (multicollinearity was not a concern given the low variance inflation factor). Models 1 and 2 examined associations of the SES index (continuous and categorical, respectively) with smoking intensity among smokers, and Models 3 and 4 with smoking history among all participants. All reported *p*-values are two-sided. Statistical analyses were performed using R 3.4.2 statistical package (GNU General Public License).

## 3. Results

Participants were, on average, 52 years old, and 64% were women. Participants reported a wide range of household income and educational attainment, with 44% having completed university education. Approximately three-quarters of participants were employed full-time, with women more likely to hold part-time employment than men. Overall, 8% and 37% were current or past smokers, respectively (Table 1). From the PCA, we retained two components with the highest eigenvalues, which explained 61% of the variation (Table 2). Component 1 included income adequacy, employment status and gender, and accounted for 35% of the variation. The positive loadings for part-time employment and for women indicate that women are more likely to hold part-time jobs. Similarly, those with full-time jobs are more likely to have higher income adequacy. Component 2, which included years of schooling, occupational prestige and income adequacy, accounted for 26% of the variation, with all positive loadings, suggesting that these variables vary together. Years of schooling and occupational prestige had the largest loadings, suggesting they had the strongest correlation with the SES index. The SES index for each individual was created by averaging the PCA scores of the two retained components. The index showed a relatively normal distribution with the mean, median and standard deviation being 0, −0.06 and 0.40, respectively.

**Figure 1:**
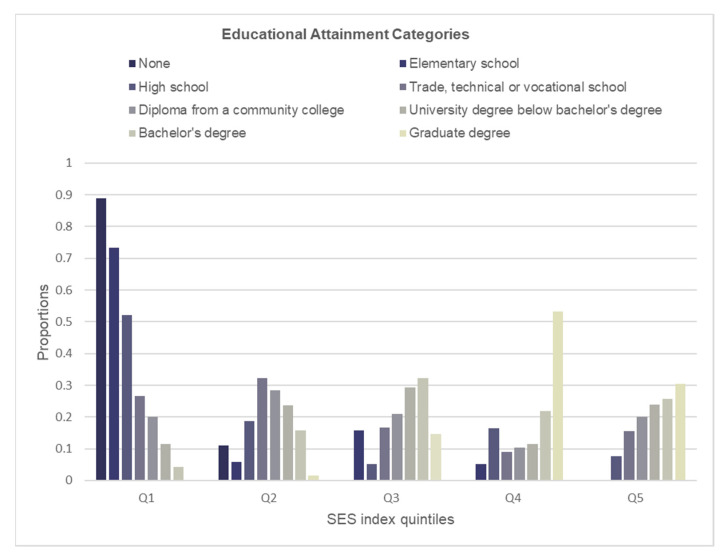
Distribution of educational attainment within each quintile of the SES index.

**Figure 2:**
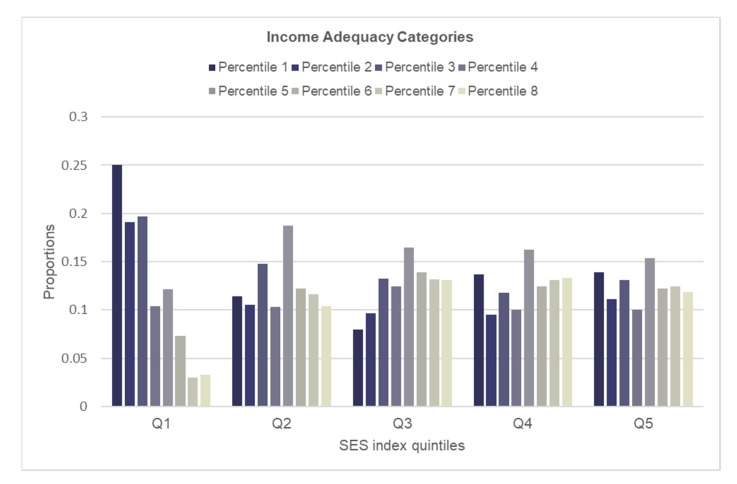
Distribution of income adequacy within each quintile of the SES index.

**Figure 3:**
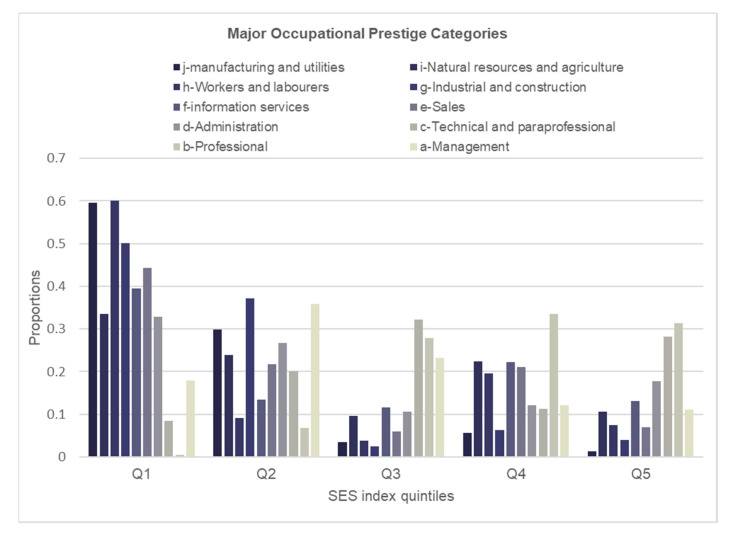
Distribution of major occupational prestige categories within each quintile of the SES index.

To assess how well the SES index aligned with individual socioeconomic indicators, we examined the distributions of educational attainment, income adequacy and occupational prestige for each quintile of the SES index (Figure 1, Figure 2 and Figure 3). Participants with lower educational attainment (i.e., high school or less) were most prevalent in the lower SES quintiles and were least prevalent in the higher SES quintiles (Figure 1). In contrast, the largest proportions of those with university education (i.e., bachelor’s or graduate degree) were found among the higher SES quintiles and the lowest proportions were among the lower SES quintiles. While the education gradient was most consistent for the lowest (1) and higher (4, 5) SES quintiles, it was most striking within the lowest SES quintile. Similar to education, a consistent and striking gradient in income adequacy was observed in the lowest SES quintile; however, the pattern for the remaining quintiles was less consistent (Figure 2). The distribution of major occupational prestige categories within each SES index quintile showed consistent patterns, except for quintile 2 (Figure 3). The gradient in the occupational ranking was most striking in the lowest and the highest quintiles.

To demonstrate the index’s utility, we examined the SES index as a continuous (Model 1) and a categorical variable (Model 2) in relation to a chronic-disease risk factor (i.e., smoking intensity). Model 1 passed the following five validation criteria: (1) it had an overall KMO of 0.54; (2) the variable-specific KMOs were above 0.52; (3) the SES index was normally distributed; (4) Bartlett’s test of sphericity test rejected the null hypothesis of no redundancy at the level of 5% (*p*-value < 0.0001); and (5) the results of multiple regression models showed that, when adjusted for age and gender, the SES index was negatively associated with smoking intensity and smoking history (*p*-value < 0.001) (Table 3). Specifically, Model 1 showed that for every 1 unit increase in the index, smoking intensity among smokers decreased, on average, by 3.86 pack-years (95% CI = −4.50, −3.21). Coefficients for Model 2 ranged from 2.16 for Q2 vs. Q1 to 4.21 for Q4 vs. Q1. Similarly, Models 3 and 4 based on smoking history among all participants showed that the SES index was negatively associated with the likelihood of being a current/past smoker (Table 3). Models 2 and 4 show a consistent and graded association of the SES index with smoking intensity among smokers and smoking history among all participants, with larger reductions observed among participants in each successive SES quintile compared to those in the lowest SES quintile.

## 4. Discussion

In this study of a large population-based sample of adults, we constructed an individual-level SES index that incorporated a set of socioeconomic and demographic indicators including years of schooling, income adequacy, occupational prestige, employment status and gender. The SES index explained 61% of the variation and was best aligned with educational attainment and occupational prestige, and to a lesser extent with income adequacy. The derived index was applied by examining its association with smoking intensity, a well-established risk factor for chronic disease. We observed a negative and graded association between the derived SES index and smoking intensity, suggesting that as SES increases, smoking intensity decreases.

Study findings highlight the utility of an index comprised of individual-level SES indicators such as years of education, income adequacy, occupational prestige and gender to achieve a global measure. As SES is a multidimensional concept, there is no single indicator that best captures an individual’s SES for all study goals and is applicable at all life course time-points in all settings—instead each individual indicator measures different, often related aspects of socioeconomic stratification and may be more or less relevant to different health outcomes and at different stages in the life course [12]. Thus, proxies for SES based on a single indicator, such as income or education which are commonly used [12,13] to capture an individual’s SES may exclude important socioeconomic information. Sometimes more than one SES indicator is included in analyses but this can lead to complex interpretation. For example, income and education present two different aspects of SES but are not interchangeable [31]; therefore, they may yield opposing trends or gradients. These limitations of using a single indicator of SES or multiple separate indicators are mitigated by creating an SES index that incorporates multiple aspects of SES into a single measure. In addition, when the goal is to adjust for socioeconomic circumstances, SES indices can better control for confounding compared to individual indicators as they offer a more comprehensive measure of SES [12].

Several SES indices have been previously developed using individual-level indicators. Chief examples include the Blishen scale, the Pineo-Porter prestige scale, The British Registrar General’s Classification, and the Hollinsgshead index [21,32,33,34]. More recent examples include Goyder and Frank’s scale of occupational prestige and Statistics Canada’s National Longitudinal Survey of Children and Youth (NLSCY) SES measure [20,24]. These indices are outdated now, and contemporary indices that go beyond education and income, and incorporate aspects such as income adequacy, occupational prestige and employment status, and include personal characteristics such as gender are needed to better capture the differences between individuals and groups in the possession of resources [35]. Although several prominent indices have successfully incorporated occupational classification to capture individuals’ SES, these have limited utility in modern epidemiologic research since they are based on outdated occupational classifications. For example, the Blishen scale assigns SES codes to occupations listed in the 1981 Canadian Classification and Dictionary of Occupations, in which indicators were derived from education and income levels for each occupational category [36]. Similarly, the Pineo-Porter method assigns prestige scores to 16 occupational categories, derived from the 1971 Census of Canada [21]. Most of the available SES indices have not incorporated gender into their construction; therefore, they may not capture inequalities in working and living conditions [16]. Since women are disproportionately more likely than men to live in poverty (a concept known as the “feminization of poverty” [37]), SES indices that incorporate gender may better capture individual’s socioeconomic circumstances and may help explain health inequities. Taken together, there is a need for an updated SES index that is derived using contemporary data and that incorporates a broader set of SES indicators beyond education and income.

Concomitant with the decline in the use of individual-level SES indices, area-level SES indices have gained popularity in epidemiologic research in the last 20 years. For example, the Pampalon and the Can-Marg indices utilise Canadian Census data to characterise residential areas in terms of several geographic characteristics (e.g., % without high school diploma; average income; % employed, % living alone; % separated, divorced, widowed; % single-parent families) [17,38]. Similarly, in the United States, the Social Deprivation Index (SDI) has been developed using data from the American Community Survey (ACS), with indicators such as % living in poverty, % with less than 12 years of education, % single parent household, % living in rented housing unit, % living in overcrowded housing unit, % of households without a car, and % non-employed adults under age 65 [39]. While some researchers may opt to use an area-level SES as a proxy for individual-level SES in the absence of individual-level data, studies have found low agreement between individual- and area-level measures [40,41]. Additionally, these indices are prone to ecological fallacy, whereby inferences about individuals are made utilizing group (i.e., area-level) characteristics. Although area-level SES indices should not be used as a substitute for individual-level SES, they nonetheless provide important population-level information. This information may be especially valuable for investigations focused on area-level characteristics (e.g., when assessing associations between aspects of the built environment and health outcomes it may be important consider area-level SES as a confounding variable).

The impact of social inequalities on health is an important topic in epidemiology, and considering SES, which is commonly measured by education and income and less frequently by occupation [42], it is critical. It is important to recognise that the influence of SES indicators may differ across settings and populations; and the importance of context when studying the influence of SES on health should not be overlooked. Our findings indicate that education is a key indicator of SES among adults from Alberta, Canada. We speculate that this finding emerged because some Albertans in occupations with lower prestige have lower education yet earn higher incomes. This is due to the primary industries dominant in Alberta’s economy. The oil and gas sector remains Alberta’s largest industry, accounting for 16% of the province’s GDP [43]. Thus, using only income and/or occupation to capture SES may not yield the expected gradient in chronic disease risk factors or outcomes. Other researchers also have emphasised the importance of setting and population. Darin-Mattsson et al. (2017) conducted a study in which an SES index was created using education, social class, occupation and income. This index was validated by testing associations between SES and limitations in daily living and psychological distress, which are health outcomes relevant to the elderly population. Income was the strongest indicator for late-life health, accounting for 3–18% of the model fit, while education contributed 0-3%, indicating that income explains more variation in late-life health [44]. In contrast, our findings indicate that income was the least consistent predictor of participants’ SES, while educational attainment exhibited the most robust gradient with SES among Albertans. 

### Strengths and Limitations

The large sample of the general adult population of Alberta with a diverse range of socioeconomic and demographic characteristics, and little missing data due to rigorous quality control measures are major strengths. Several limitations warrant consideration. Data on socioeconomic and demographic indicators, smoking and confounders were collected through self-report, which is prone to measurement error and social desirability bias. However, self-report is the only feasible method of collecting this type of information in large population-based studies. The validity of self-reported smoking is consistently high in population-based studies [45], however the validity of self-reported cigarette use used to construct smoking intensity is not known. With regard to educational attainment, there is no possibility of confirming whether the education, particularly post-secondary education, was received in Canada, which likely affects income and occupational prestige scores [46]. Although participants self-reported their occupation using an open-ended question, misclassification of occupational classes was possible when assigning NOC codes. While research on SES inequalities in health often relies on occupational prestige scores [47,48], the occupational prestige scores for Canada are dated and are in need of revision. Despite considering a comprehensive set of commonly used indicators to construct the SES index and assess its utility, residual confounding may persist despite adjustment due to the lack of information on other risk factors and chronic diseases. Generalisability of the findings to populations outside Alberta and/or Canada may be limited, however future studies would be able use the process described in this paper to construct SES indices that are generalisable to their study populations. Finally, since we used secondary data, we were limited to using a binary variable measuring gender. As a result, the diversity in gender was not captured and this could have led to gender misclassification. Future population-based studies should include transgender and non-binary individuals and utilise measurements that recognise gender diversity, although such studies may lack statistical power to conduct gender-specific analyses given the small proportions of the general population these individuals represent [49].

## 5. Conclusions

In this study, we created an SES index using a comprehensive set of common indicators in epidemiologic research. We examined how well the derived SES index aligned with its main components: educational attainment, household income, and occupational prestige, and assessed its association with smoking intensity. An updated, more comprehensive and encompassing SES index is needed to facilitate our understanding of the SES gradient in chronic disease risk factors and outcomes. Yet, in recent years, researchers’ attention has been focused on developing area-level indices. Using the process we described in this paper, future research studies will be able to derive an individual-level SES index when assessing socioeconomic inequalities in relation to a range of chronic disease risk factors as well as chronic disease outcomes.

## Figures and Tables

**Table 1:** Characteristics of participants that completed the Core Questionnaire.

Characteristic	Total (*n* = 17,371)		Men (*n* = 6188)		Women (*n* = 11,183)	
Age, mean SD	52.01	8.59	53.24	8.88	51.33	8.36
Age, *n*%						
35–44	4024	23.17%	1264	20.43%	2760	24.68%
45–54	6569	37.82%	2137	34.53%	4432	39.63%
55–64	5689	32.75%	2208	35.68%	3481	31.13%
≥65	1089	6.27%	579	9.36%	510	4.56%
Educational attainment, *n*%						
None or elementary School	182	1.05%	91	1.47%	91	0.82%
High School	3109	17.90%	1035	16.73%	2074	18.55%
Trade, technical or vocational school	2437	14.03%	1413	22.83%	1024	9.16%
Diploma from a community college	4082	23.50%	951	15.37%	3131	28.00%
University degree below bachelor’s	766	4.41%	210	3.39%	556	4.97%
Bachelor’s Degree	4463	25.69%	1507	24.35%	2956	26.43%
Graduate Degree	2332	13.42%	981	15.85%	1351	12.08%
Years of schooling, mean SD	14.97	2.66	15.17	2.78	14.86	2.58
Household income, *n*%						
<$10,000	57	0.33%	10	0.16%	47	0.42%
$10,000–$24,999	407	2.34%	87	1.41%	320	2.86%
$25,000–$49,999	1680	9.67%	395	6.38%	1285	11.49%
$50,000–$74,999	2568	14.78%	779	12.59%	1789	16.00%
$75,000–$99,999	2880	16.58%	1039	16.79%	1841	16.46%
$100,000–$149,999	4667	26.87%	1786	28.86%	2881	25.76%
$150,000–$199,999	2643	15.22%	1025	16.56%	1618	14.47%
≥$200,000	2469	14.21%	1067	17.24%	1402	12.54%
Region, *n*%						
Rural	14,334	82.52%	5088	82.22%	9246	82.68%
Urban	2812	16.19%	1005	16.24%	1807	16.16%
Outside Alberta	225	1.30%	95	1.54%	130	1.16%
Marital status, *n*% *						
Married/living with a partner	13,399	77.16%	5256	85.01%	8143	72.82%
Divorced	1808	10.41%	371	6.00%	1437	12.85%
Widowed	418	2.41%	79	1.28%	339	3.03%
Separated	494	2.84%	122	1.97%	372	3.33%
Single, never married	1246	7.18%	355	5.74%	891	7.97%
Family structure, *n*% *						
Couple with children	3729	21.54%	1495	24.27%	2234	20.04%
Couple no children	6674	38.56%	2576	41.81%	4098	36.76%
Single parent	471	2.72%	84	1.36%	387	3.47%
Extended family	4032	23.29%	1398	22.69%	2634	23.63%
Living alone	2404	13.89%	608	9.87%	1796	16.11%
Occupational prestige, *n*%						
Management	3069	17.67%	1554	25.11%	1515	13.55%
Professional	5440	31.32%	1733	28.01%	3707	33.15%
Technical and paraprofessional	1962	11.29%	680	10.99%	1282	11.46%
Administration	3062	17.63%	323	5.22%	2739	24.49%
Sales	872	5.02%	342	5.53%	530	4.74%
Information services	1363	7.85%	264	4.27%	1099	9.83%
Industrial and construction	676	3.89%	619	10.00%	57	0.51%
Workers and labourers	481	2.77%	357	5.77%	124	1.11%
Natural resources and agriculture	218	1.25%	130	2.10%	88	0.79%
Manufacturing and utilities	228	1.31%	186	3.01%	42	0.38%
Employment status, *n*% *						
Full-time	12,908	74.31%	5360	86.62%	7548	67.50%
Part-time	4609	26.53%	867	14.01%	3742	33.46%
Other	2117	12.19%	443	7.16%	1674	14.97%
Smoking history, *n*% *						
Current	1410	8.12%	522	8.44%	888	7.94%
Past	6464	37.21%	2264	36.59%	4200	37.56%
Never	9455	54.43%	3385	54.70%	6070	54.28%
Smoking intensity (pack-years), mean SD *						
Smokers	10.57	11.02	12.65	12.47	9.44	9.98
All participants	4.56	8.93	5.38	10.26	4.11	8.08

SD: standard deviation; * may not add up to 17,371 due to missing values or participants being able to choose more than one answer.

**Table 2:** PCA loadings for two components included in the SES index.

	Component 1	Component 2
Years of schooling	-	0.77
Occupational prestige	-	0.79
Income adequacy	−0.2	0.57
Employment status (full-time)	−0.97	-
Employment status (part-time)	0.97	-
Gender (women)	0.4	-

**Table 3:** Estimated associations of the SES index with smoking intensity (pack-years) and smoking history (past/current vs. never).

	Smoking Intensity		Smoking History	
	Coefficient ^a^ (95% CI)	SE	Coefficient ^b^ (95% CI)	SE
	Model 1		Model 3	
SES index	−3.86 (−4.50, −3.21)	0.33	−0.80 (−0.88, −0.72)	0.04
Gender (ref: men)	−1.40 (−1.94, −0.86)	0.28	0.28 (0.22, 0.36)	0.03
Age	0.32 (0.29, 0.35)	0.016	0.04 (0.04, 0.04)	<0.01
	**Model 2**		**Model 4**	
SES index quintiles (ref: Q1)				
Q2 vs. Q1	−2.16 (−2.87, −1.45)	0.36	−0.29 (−0.38, −0.20)	0.05
Q3 vs. Q1	−3.01 (−3.77, −2.26)	0.39	−0.65 (−0.75, −0.56)	0.05
Q4 vs. Q1	−3.22 (−3.99, −2.45)	0.39	−0.78 (−0.88, −0.69)	0.05
Q5 vs. Q1	−4.21 (−5.00, −3.42)	0.40	−0.89 (−0.99, −0.79)	0.05
Gender (ref: men)	−1.47 (−2.01, −0.93)	0.28	0.29 (0.22, 0.35)	0.03
Age	0.31 (0.28, 0.34)	0.016	0.04 (0.03, 0.04)	<0.01

SE: standard error; CI: confidence interval; SES: socioeconomic status; ref: reference category; Q: quintile. ^a^ Derived from multiple linear regression of smoking intensity (pack-years) as the outcome, adjusted for age and gender. ^b^ Derived from multiple logistic regression of smoking history (past/current smoker vs. never) as the outcome, adjusted for age and gender.

## Data Availability

Access to individual-level data is available in accordance with the Health Information Act of Alberta and Alberta’s Tomorrow Project (ATP). Access Guidelines at https://myatpresearch.ca (accessed on 28 March 2022).
